# Care services for elderly people with dementia in rural China: a case study

**DOI:** 10.2471/BLT.15.160929

**Published:** 2016-01-06

**Authors:** Christina Wu, Lin Gao, Shulin Chen, Hengjin Dong

**Affiliations:** a Center for Health Policy Studies, Zhejiang University School of Medicine, Zijingang Campus, 866 Yuhangtang Rd, Hangzhou, 310058 China.; b Department of Psychology, Zhejiang University, Hangzhou, China.

## Abstract

**Objective:**

To determine the state of the health and supportive services available to elderly people with dementia – and their families – in rural Lanxi county, in the province of Zhejiang, China.

**Methods:**

In November 2014 and January 2015, we interviewed 14 key informants on dementia care face-to-face, using a semi-structured questionnaire. The informants included three rural physicians, an urban geriatrician, seven directors of institutions for the care of the elderly and three officials of the civil affairs bureau. We also completed in-depth interviews with five family caregivers of elderly people with dementia.

**Findings:**

The interviewees indicated that there was a lack of specialized services designed specifically to address the needs of individuals with dementia and their family members. Non-psychiatric medical services and the available facilities for long-term care appeared to be ill-equipped to manage these needs. They lacked both clinical staff and standardized, evidence-based practices for the diagnosis, care, treatment and rehabilitation of patients with dementia. As care facilities often refused to admit elderly people with dementia, families were generally forced to care for elderly relatives with dementia at home.

**Conclusion:**

In Lanxi county – and probably in much of rural China – more public resources are needed to support family caregivers and to improve the capacity of care facilities for the elderly to care for individuals with dementia.

## Résumé

**Objectif:**

Déterminer la situation en matière de services médicaux et de services d’aide proposés aux personnes âgées atteintes de démence – et à leurs familles – dans le comté rural de Lanxi, province de Zhejiang, en Chine.

**Méthodes:**

En novembre 2014 et janvier 2015, nous avons interrogé en face à face 14 informateurs clés au sujet de la prise en charge des personnes atteintes de démence, à l'aide d'un questionnaire semi-directif. Ces informateurs comprenaient trois médecins de campagne, un gériatre de ville, sept directeurs d'institutions accueillant des personnes âgées et trois fonctionnaires du bureau des affaires civiles. Nous nous sommes également entretenus en détail avec cinq aidants familiaux s'occupant de personnes âgées atteintes de démence.

**Résultats:**

Les personnes interrogées ont fait part de l'absence de services spécialisés permettant de répondre aux besoins des personnes atteintes de démence et de leurs proches. Il est apparu que les services médicaux non psychiatriques et les structures de soins de longue durée disponibles étaient mal outillés pour répondre à ces besoins. Ils manquaient autant de personnel clinique que de pratiques standardisées reposant sur l'expérience pour le diagnostic, la prise en charge, le traitement et la rééducation des patients atteints de démence. Comme les établissements de soins refusaient souvent d'admettre les personnes âgées atteintes de démence, les familles étaient généralement obligées de s'en occuper à la maison.

**Conclusion:**

Le comté de Lanxi – et probablement de nombreuses autres régions de Chine rurale – nécessite davantage de ressources publiques pour soutenir les aidants familiaux et renforcer la capacité des établissements accueillant des personnes âgées à prendre en charge les personnes atteintes de démence.

## Resumen

**Objetivo:**

Determinar el estado de los servicios de salud y de apoyo disponibles para las personas mayores con demencia (y sus familias) en el condado rural de Lanxi, en la provincia de Zhejiang, China.

**Métodos:**

En noviembre de 2014 y enero de 2015 se entrevistó a 14 informadores clave sobre el cuidado de la demencia de forma presencial, utilizando un cuestionario semiestructurado. Entre los informadores se encontraban tres médicos rurales, un geriátrico urbano, siete directores de instituciones de cuidados a las personas mayores y tres empleados públicos de la oficina de asuntos civiles. También se realizaron entrevistas a fondo con cinco cuidadores familiares de personas mayores con demencia.

**Resultados:**

Los entrevistados indicaron que había una falta de servicios especializados diseñados especialmente para tratar las necesidades de individuos con demencia y sus familiares. Los servicios médicos no psiquiátricos y las instalaciones disponibles para cuidados a largo plazo no parecían estar bien pertrechados para abordar estas necesidades. Carecían de personal clínico y prácticas estandarizadas basadas en pruebas para el diagnóstico, cuidado, tratamiento y rehabilitación de pacientes con demencia. Puesto que las instalaciones de atención solían rechazar a personas mayores con demencia, las familias normalmente se veían obligadas a cuidar de sus parientes ancianos con demencia en sus hogares.

**Conclusión:**

En el condado de Lanxi (y probablemente en gran parte de la China rural) se necesitan más recursos públicos para apoyar a los cuidadores familiares y mejorar la capacidad de las instalaciones de atención para las personas mayores, de modo que puedan tratar a personas con demencia.

## ملخص

**الغرض:**

الوقوف على الحالة الصحية والخدمات الداعمة المتاحة لكبار السن المصابين بالخَرَف (تدهور القدرات الذهنية) وأسرهم في مقاطعة "لانشي" الريفية في إقليم "تشجيانغ" بالصين.

**الطريقة:**

في نوفمبر/تشرين الثاني من عام 2014 ويناير/كانون الثاني من عام 2015، قمنا بإجراء مقابلات مع 14 من المبلّغين الرئيسيين عن رعاية الخَرَف مباشرةً، وذلك باستخدام استبيان شبه مقنن. وتضمنت مجموعة المبلّغين ثلاثة أطباء ريفيين، واختصاصيًا في أمراض الشيخوخة بالمناطق الحضرية، وسبعة مدراء في مؤسسات لرعاية كبار السن، وثلاثة مسؤولين من مكتب الشؤون المدنية. كما قمنا أيضًا باستكمال مقابلات متعمقة مع خمسة من عائلات مقدمي الرعاية الصحية لكبار السن المصابين بالخَرَف.

**النتائج:**

أشار الأشخاص الذين جرت مقابلتهم إلى وجود نقص في الخدمات المتخصصة المصممة خصيصًا لتلائم احتياجات المصابين بالخَرَف وأفراد أسرهم. وبدت الخدمات الطبية غير النفسية والمرافق المتاحة للحصول على الرعاية طويلة الأمد غير مؤهلة لملاءمة هذه الاحتياجات، فقد كانت تفتقد إلى وجود الطاقم الطبي والممارسات المعيارية والمبنية على الشواهد لأغراض التشخيص وتقديم الرعاية والعلاج وإعادة التأهيل للمرضى المصابين بالخَرَف. ونظرًا لأن مرافق الرعاية الصحية غالبًا ما ترفض قبول علاج كبار السن المصابين بالخَرَف، فقد أجبرت الأسر بشكل عام على رعاية الأقارب المسنين المصابين بالخَرَف في المنزل.

**الاستنتاج:**

في مقاطعة "لانشي" – وربما في العديد من المناطق الريفية في الصين – هناك حاجة إلى المزيد من الموارد العمومية لدعم مقدمي الرعاية من بين أفراد الأسرة وتحسين قدرة مرافق رعاية كبار السن لرعاية المصابين بالخَرَف.

## 摘要

**目的:**

旨在确定——中国浙江省兰溪县农村——痴 呆老人的健康状态及其家人享有的支持服务。

**方法:**

2014 年 11 月至 2015 年 1 月，我们利用半结构式 问卷面对面访问了 14 名痴呆护理方面的关键受访者。此受访者包括三名农村医师、一名城市老年病学专家、

七名养老院主任和三名民政局的办事人员。我们还对 五名老年痴呆的家庭护理人员进行了深度访问。

**结果:**

此受访者指出缺乏专门针对满足痴呆老人和其家 庭成员需求的专业服务。非精神科医疗服务和长期护 理的有效机构在管理这些需求时貌似装备不良。他们 缺乏临床人员和标准、基于证据的实践，以诊断、护理、治疗和康复痴呆患者。由于养老院经常拒绝接纳老年 痴呆症患者，其家人通常被迫在家护理痴呆老人。

**结论:**

兰溪县——或中国大部分农村地区——需要更多 的公共资源支持家庭护理人员和提升养老院护理痴呆 老人的能力。

## Резюме

**Цель:**

Определить состояние медико-санитарных служб и поддерживающих услуг, доступных пожилым людям, страдающим от деменции, и их родным, которые проживают на сельской территории уезда Ланьси, провинция Чжэцзян, Китай.

**Методы:**

В ноябре 2014 года и январе 2015 года был проведен опрос 14 ключевых информаторов, предоставляющих информацию по уходу за людьми с деменцией, который осуществлялся при очной встрече с помощью полуструктурированного интервью. В число информаторов входили три сельских врача, городской врач-гериатр, семь руководителей учреждений, предоставляющих услуги по уходу за престарелыми, и три должностных лица из бюро по гражданским вопросам. Были также проведены содержательные интервью с пятью лицами, осуществляющими уход за престарелыми людьми с деменцией, которые являются членами их семей.

**Результаты:**

Опрошенные лица отметили недостаток специализированных услуг, предназначенных для удовлетворения потребностей лиц, страдающих от деменции, и членов их семей. Медицинские службы непсихиатрического профиля и доступные учреждения для долгосрочного ухода оказались недостаточно оборудованы для удовлетворения этих потребностей. Нехватка персонала в них сочеталась с недостатком стандартизированных, основанных на опыте практик для диагностики, осуществления ухода, лечения и реабилитации пациентов с деменцией. Поскольку организации по уходу часто отказывались принимать на лечение пожилых людей с деменцией, их семьям обычно приходилось самим обеспечивать уход за ними дома.

**Вывод:**

В уезде Ланьси и, скорее всего, в большинстве сел Китая требуются дополнительные государственные ресурсы для поддержки лиц, осуществляющих уход на дому, или для увеличения возможностей организаций по уходу за пожилыми людьми осуществлять помощь в обслуживании людей с деменцией.

## Introduction

In China, the longevity of the inhabitants and the prevalence of conditions associated with ageing are increasing. Between 2010 and 2040, the proportion of the population that is 65 years or older is expected to more than double from 9.0% to 22.6%.^1^ Since the 1980s, family-planning policies and a decline in births have led to reductions in the number of working-age people.^2^ The old-age dependency ratio – i.e. the number of people that are 65 years or older per 100 people aged 20 to 64 years – is expected to increase from 13 in 2010 to 45 in 2050.^3^

The global number of people with dementia –i.e. degenerative brain diseases characterized by the progressive loss or decline of memory and other cognitive abilities – is increasing in most countries.^4^ In 2014, the prevalence of dementia in China was estimated – from five representative centres – to be 5.1% (528/10276) and 23.3% (96/412) among individuals older than 64 years and 84 years, respectively.^5^ The estimated number of people with dementia increased from 3.68 million in 1990 to 9.19 million in 2010.^6^ The condition not only reduces the earnings of the people with dementia and/or their families – by an estimated mean of 1159 yuan (¥) or about 183 United States dollars (US$) per month per person with dementia – but also adds an estimated ¥51.3–59.8 billion to the national health-care costs annually.^7^

The care and support of the elderly is traditionally a familial responsibility.^8^ As a result, admission to facilities for the care of the elderly – which was highly stigmatized – has generally been restricted to individuals who were unable to work and did not have a source of income or any legal guardians. Until recently, the government has not provided further options for the care of the elderly.^3^

Demographic shifts and socioeconomic changes are now weakening the tradition of familial care of the elderly. In cities, the family structure often consists of four grandparents, two parents and one child.^9^ Many elderly people are choosing to live separately from their adult children^10^ and migration of young workers to urban areas and historical shifts in a family’s welfare function have eroded family tradition in many rural settings.^11^ Faced with these trends, the government aims to establish three tiers of services for the elderly: (i) home-based care as the basis; (ii) community-based services as support; and (iii) institutional care as the last resort. However, the home- and community-based services that are available are limited.^3^ Although policy inducements have promoted the development of institutions for the care of the elderly^3^ and institutional care appears to have become more acceptable to the elderly and their adult children,^12^ the capacity to provide such care lags behind the need. Institutions for the care of the elderly rarely have clinical staff and most of their employees are rural migratory workers,^1^^,^^13^ who lack the training needed to manage the behavioural and psychological symptoms of dementia.^13^^,^^14^ In consequence, many institutions simply refuse to admit anyone with dementia or, at least, advanced dementia. A study in Chengdu found that of the 10 institutions that they investigated, five rejected people with advanced dementia.^13^ Home- and community-based dementia-specific services – e.g. day-care, respite, caregiver support and case management – only exist in the Hong Kong Special Administrative Region, Taiwan and a few major urban centres.^15^

One result of the general scarcity of dementia-specific services^16^ is that little is known about the care of people with dementia in medical, institutional and community-based settings in China. The main aim of the present study was to use information collected in interviews in Lanxi county, to determine the current state of the health and supportive services available to elderly people with dementia – and their families – in rural areas.

## Methods

### Study site

Lanxi county, which covers 1310 km^2^ and had a population of about 666 000 in 2014, is located in the mid-west of the eastern coastal province of Zhejiang^17^ and falls under the administration of Jinhua prefecture. We specifically selected Lanxi as our study site because it lies in a province which has encountered serious problems caused by an increased ageing population,^18^ and we had previously collaborated with the county’s health administrators. In 2010, people that were 65 years or older accounted for about 12.6%, 11.2% and 8.9% of the populations of Lanxi, Zhejiang and China, respectively.^19^^–^^21^ Since very few community-based or institutional-care services for elderly individuals with dementia exist anywhere in rural China, we expected that our findings would reflect the situation in most rural settings in the country.

### Data collection and analysis

We collected relevant data during key-informant interviews and site visits conducted in November 2014 and January 2015 (Table 1). The 14 key informants were comprised of two rural physicians in the administrative centre of the county, an urban geriatrician from the geriatric hospital in the administrative centre of the county, a rural physician from one of the study villages, seven directors of institutions for the care of the elderly and three officials of the civil affairs bureau. We visited and made observations at seven facilities (Table 1). During the visits, we were given a tour of the facility, conducted informal interviews with facility staff (e.g. care staff and clinical staff) and took notes and photographs of pre-specified categories (e.g. residential facilities for people with dementia, health-care professionals, information infrastructure and management). At the end of each day, the team members would write a report about the day’s observations. We also conducted in-depth interviews, in the caregivers’ homes, with five family caregivers of individuals with dementia identified by local physicians. Three of these caregivers lived in the county seat and the others in two different villages. We employed maximum variation sampling and selected institutions and interviewees purposively to represent a wide range of variation in the factors of interest – e.g. geographical context, price level and socioeconomic status of the family caregivers.

**Table 1:** Study facilities in Lanxi county, China, 2014

Type of facility	Setting	No. of facilities		
		With interviewed staff member(s)^a^	Visited	Total
Geriatric hospital	Urban	1	1	1
Township hospital	Urban	1	1	1
Village clinic	Rural	1	1	1
Government-owned institution for long-term care	Rural	1	1	1
	Urban	1	1	1
Private institution for long-term care	Urban	3	2	3
Day-care centre for adults	Urban	2	1	2

^a^ In some instances the interviewees went to another facility to be interviewed.

All interviews were conducted by a member of the research team using a semi-structured interview guide. Interviews lasted 30 to 60 minutes and were recorded with a digital voice recorder. All but three interviews were conducted in Mandarin; the exceptions were interviews with three family caregivers, which had to be conducted in the local Lanxi dialect, using an interpreter. Using the audio recordings, each interview was simultaneously transcribed and translated into English. Transcripts were coded for general topics used to guide the interviews and codes were continually refined during the coding process. One researcher coded all of the data and a second researcher reviewed a subset of the coding. Findings were considered in relation to the World Health Organization Innovative Care for Chronic Conditions Framework.^22^

Written informed consent was obtained from the family caregivers. No reimbursements were given to the interviewees. The study protocol was approved by the School of Public Health at the Institutional Review Board of Zhejiang University.

## Results

### Medical services

In Lanxi, a geriatric hospital and the county hospital offer both inpatient and outpatient services. At the time of our study, the 50-bed geriatric hospital lacked standardized procedures for the diagnosis or treatment of dementia and only addressed related physical problems – e.g. pneumonia and injuries caused by falls. The geriatrician, who worked in this hospital, said that:

“Early diagnosis may help to slow progression [of dementia] but we have no standards or motivation to deal with dementia since it is the responsibility of psychiatric hospitals.”

The urban township hospital we investigated did not have any staff specializing in the diagnosis and treatment of neuropsychiatric disorders and the staff were unable to prescribe medications for dementia. Any patients who needed such diagnosis, treatment or medications were referred to a psychiatric hospital. The medications used to treat dementia were relatively expensive and were not always covered by health insurance plans.

The three interviewed rural physicians were older than 60 years and had received short-term training in basic health care to become village doctors.^23^ They appeared to have poor knowledge of – and skills in – dementia diagnosis and management. They did not use any formal screening instruments for diagnosing dementia, could only identify ageing as a cause and were not aware of any treatments. They did not provide the family of an individual with suspected dementia with information about additional services or care advice. The following quote reflects the general attitude of village physicians towards dementia:

“In the countryside, there is a common notion that going to the doctor for this kind of illness [dementia] is a waste of money since it cannot be cured.”

### Institutions for long-term care

Characteristics of three different types of institutions we visited for long-term care are presented in Table 2.

**Table 2:** Characteristics of three different types of institutions for long-term care in Lanxi county, China, 2014

Characteristic	Institution		
	Rural area	Urban area	
	Government-owned	Government-owned	Private**^a^**
No. of residents	90–100	168	140
Mean age of residents, years (range)	80 (67–94)	85 (67–103)	80 (59–104)
Monthly cost per resident, ¥			
Without dementia	1000^b^	960–2000^c^	950–1000^b^
With dementia	1100^b^	1900–3000^c^	2000^b^
Level of government subsidy	Fully funded by government	One-time subsidy from provincial government of ¥6000 per bed	One-time subsidies of ¥6000 per bed from provincial government and ¥500 per bed from local government
No. of clinical staff	0	5^d^	0
No. of care staff	13	34	20
Monthly salary of care staff, ¥	2500–3000	3000–4000	2600–3700
Education level of care staff	Illiterate or primary school	Primary school	Primary school or rarely high school
Age range of care staff, years	60–70	60–70	40–60
Care staff to resident ratio	1:7	1:5	1:7
% of people with dementia among residents^e^	30	50	20

¥: yuan.

^a^ Recorded as facility A, B and C.

^b^ Residents sleep two per room.

^c^ Cost varies depending on the number of residents per bedroom, which may be one, two or six.

^d^ Two primary-care physicians and three nurses.

^e^ As estimated by the leaderships of the facilities.

Note: The average conversion rate in 2014 was ¥1 to 0.162 US dollars.

#### Public sector

The government-owned rural institution refused to admit any elderly individuals who were reported to have dementia. However, at the time of our study, about one-third of the residents appeared to have dementia (Table 2). These residents had, presumably, either developed dementia while living in the institution or had been admitted without their families reporting that they had dementia. There were no formal medical evaluations of elderly individuals before their admission. Most of the families that had elderly relatives in the institution did not seek medical or psychiatric consultations for their relatives and left the institution’s staff to make all decisions about their relatives’ care. Most of the residents who appeared to have dementia were not taking any dementia-related medication. The institution had no clinical staff and relied on the assistance of local village physicians – who were often unavailable. Each of the 13 caregivers had been made responsible for the care of about seven residents – including one or two of those with dementia. Following complaints from other residents, the residents with dementia were boarded together.

Eight of the caregivers each slept beside an elderly resident with advanced dementia so that they could provide around-the-clock care. The institution did not provide any formal training in dementia care for staff or any rehabilitative or psychological support for the residents with dementia. The attitude of the institution’s leadership towards dementia care can be summed up by the following quote from the director:

“Since all dementia patients’ symptoms and levels of severity differ, it is difficult to be trained in general caregiving strategies since their care must be tailored to each individual patient’s idiosyncrasies and behaviours.”

The institution had recently initiated a mandatory trial period for new residents, so that those with dementia could be identified and screened out.

The government-owned urban institution was established in 1999 and had some medical and nursing capacity. Every individual seeking admission is subjected to a formal medical evaluation and those found to have dementia are rarely admitted. However, many residents develop dementia and this is diagnosed by staff based on the residents’ speech, obvious lapses in memory and inability to recognize family members. Residents with dementia were housed in a separate part of the institution. The facility did not offer medication, psychosocial interventions or rehabilitation. At the time of our study, the doctors who worked in the institution were all retired primary-care physicians. Although several of the institution’s caregivers had attended training on basic dementia care, most lacked the literacy that might enable them to benefit from more advanced training. The institution’s director stated that “We cannot handle more people with advanced dementia simply because we have limited resources in staffing and energy.”

#### Private sector

The estimated proportion of residents with dementia varied greatly in the three private-sector institutions that we investigated – which we recorded as facilities A, B and C (Table 2). Although all three institutions charged more for the care of a resident with dementia than for the care of another resident, none of them provided medical, psychological or rehabilitative support for dementia beyond extra staff chatting with the residents with dementia. According to facility B’s director, facility staff could only manage the symptoms of dementia and provide extra management. Facility C’s director sent staff to nearby cities for training in person-centred care. Interestingly, most of the caregivers in all three institutions were older than 60 years and educated only to primary-school level. Facility A’s director had recently attracted younger and more educated caregivers by offering higher salaries.

### Day-care centres

Local government provides day-care centres for adults – i.e. community-based recreational spaces for meals and group activities – but participant fees have to cover these centres’ operating costs. The urban day-care centre that we visited was staffed by three attendants. All 40 elderly people served by the centre were older than 80 years and had no obvious disability. They each paid ¥4 (US$ 0.63) per day to cover the operating costs and two meals. Rural physicians offered medical services in the centre. However, this centre – like all Chinese day-care centres for adults – was designed to serve relatively healthy individuals and therefore lacked the resources to manage the needs of elderly individuals with dementia.

### Home-based care

Since systems of home-based care for the elderly differed with the socioeconomic status of the household, we interviewed family caregivers from households that had low, moderate and high per-capita incomes.

High-income households could afford to hire a live-in caregiver for about ¥3000 (US$ 473) per month. Such caregivers are usually low-skilled rural migratory workers who can address the physical needs of elderly people with dementia but lack specialized training in dementia care. One interviewed wife of a man with dementia had opted to pay her two daughters to provide care for their father.

Most middle-income households cannot afford to hire a caregiver. In such households it is often a spouse – who is still in good health – who cares for an individual with dementia, often with some small level of support from an adult child or a child’s spouse. Such households can usually afford to seek specialist consultation and acquire prescribed medications to control the symptoms of dementia.

Low-income households have few resources to support dementia care. Financial support from adult children is often negligible. Such households do not seek specialist consultation for dementia and receive only basic medical support from rural physicians.

## Discussion

To promote optimal health outcomes for the people needing long-term care, the Innovative Care for Chronic Conditions Framework suggests the formation of a health care triad – consisting of the patient and family, health care team and the community supporters.^22^ Strengthening the linkages within this triad improves care for elderly individuals with dementia, especially in rural areas. First, the health-care team can disseminate knowledge of dementia and its management to families and community partners. We found that families know little about dementia care and how medical care could help. Once their relatives are institutionalized, families assume a secondary role in care management and rarely seek medical consultations for their relatives. Second, training and tools for health-care teams need to be improved. We found that rural physicians often diagnose patients with dementia but are unaware of treatment options. Community partners – i.e. the institutions and services for long-term care – generally lack knowledge and training in providing person-centred care and other evidence-based practices for dementia care, treatment and rehabilitation. Third, the broader community and health-care organizations could support efforts to provide dementia care and reduce stigma. Resources for more community-based and home-based services, such as care managers or better-equipped adult day-care centres, need to be mobilized. Finally, a positive policy environment is also necessary for improving the care. In Lanxi county – and probably elsewhere in China – policies that incentivize the admission of elderly people with dementia to institutions for long-term care and/or the improvement of dementia-related human resources should be considered.

The costs of services for dementia care are covered by social health insurance and out-of-pocket payments.^15^ In 2003, the government launched a new cooperative medical scheme to target rural areas, where 80% of residents lacked health insurance.^24^ However, most long-term care is paid for out-of-pocket, even in government-run homes.^1^

Elderly people living in rural areas primarily receive their care from village clinics.^25^ In 2010, 92.3% of villages had clinics that were staffed with rural physicians who provided primary care. Recent policy reforms have further emphasized the primary responsibility of rural physicians in village clinics for management of chronic diseases, including dementia.^26^ In China, there are 39 904 institutions for long-term care, of which 31 472 are located in rural areas.^21^ If our results are applicable to the rest of the country, this would mean that the majority of institutions do not provide dementia-specific care. Of the institutions run by the government, the rural facilities are restricted to individuals who are unable to work and do not have a source of income and do not have any legal guardians, while the urban facilities have no such restrictions on their admissions. The private-sector institutions – which are under the regulation and supervision of the civil affairs bureau – vary greatly in costs, facilities, equipment, staffing and services according to the institutions’ leadership and funding.^1^ In general, compared with their public-sector counterparts, private-sector institutions are more likely to be understaffed and more likely to have residents who are very ill.^27^

As previous studies have shown,^16^^,^^28^ we observed that most home-based dementia care is informal and provided by family members, although it may be supplemented by formal services provided by paid caregivers. However, a study in four cities across Zhejiang province has shown that among disabled (e.g. bedridden or living with dementia for six months or longer) elderly individuals living at home, only 5.9% (26/435) of those in rural areas received any formal care – compared with 36.9% (144/391) in urban areas.^29^

Although the family is still regarded as the most reliable source of care for elderly people with dementia, this option will become much less feasible in the coming decades. More public resources are needed to support family caregivers and to improve institutional capacity – in terms of both space and skills of the staff – for individuals with dementia. Financial supplements should be offered to institutions for each resident diagnosed with advanced dementia. The government should invest in building a long-term workforce – for both institutional and home-based care – and train institutional caregivers about integrative care for dementia. Since recent policy reforms have largely emphasized rural physicians’ role in managing chronic diseases such as dementia, collaboration between rural physicians, care managers and specialists in psychiatry and neurology could lead to better outcomes for older adults with dementia. This would require a multi-level approach, involving the development of dementia training for rural physicians, the care manager as a new professional role, community-based resources for dementia care and educational campaigns to dispel common misconceptions regarding dementia.
